# Pneumococcal conjugate vaccine 13 delivered as one primary and one booster dose (1 + 1) compared with two primary doses and a booster (2 + 1) in UK infants: a multicentre, parallel group randomised controlled trial

**DOI:** 10.1016/S1473-3099(17)30654-0

**Published:** 2018-02

**Authors:** David Goldblatt, Jo Southern, Nick J Andrews, Polly Burbidge, Jo Partington, Lucy Roalfe, Marta Valente Pinto, Vasilli Thalasselis, Emma Plested, Hayley Richardson, Matthew D Snape, Elizabeth Miller

**Affiliations:** a Immunobiology Unit, UCL Great Ormond Street Institute of Child Health, London, UK; b Immunisation Hepatitis and Blood Safety Department, Public Health England, Colindale, London UK; c Statitstics, Modelling and Economics Department, Public Health England, Colindale, London UK; d Oxford Vaccine Group, Department of Paediatrics, University of Oxford, Oxford, UK

## Abstract

**Background:**

Infants in the UK were first offered a pneumococcal conjugate vaccine (PCV7) in 2006, given at 2 and 4 months of age and a booster dose at 13 months (2 + 1 schedule). A 13-valent vaccine (PCV13) replaced PCV7 in 2010. We aimed to compare the post-booster antibody response in UK infants given a reduced priming schedule of PCV13 (ie, a 1 + 1 schedule) versus the current 2 + 1 schedule and to assess the potential effect on population protection.

**Methods:**

In this multicentre, parallel group, randomised controlled trial, we recuited infants due to receive their primary immunisations aged up to 13 weeks on first vaccinations by information booklets mailed out via the NHS Child Health Information Service and the UK National Health Application and Infrastructure Services. Eligible infants were randomly assigned (1:1) to receive PCV13 at 2, 4, and 12 months (2 + 1 schedule) or 3 and 12 months of age (1 + 1 schedule) delivered with other routine vaccinations. Randomisation was done by computer-generated permuted block randomisation, with a block size of six. Participants and clinical trial staff were not masked to treatment allocation. The primary endpoint was serotype-specific immunoglobulin G concentrations values (geometric mean concentrations [GMC] in μg/mL) measured in blood samples collected at 13 months of age. Analysis was by modified intention to treat with all individuals included by randomised group if they had a laboratory result. This trial is registered on the EudraCT clinical trial database, number 2015-000817-32, and ClinicalTrials.gov, number NCT02482636.

**Findings:**

Between September, 2015, and June, 2016, 376 infants were assessed for eligibility. 81 infants were excluded for not meeting the inclusion criteria (n=50) or for other reasons (n=31). 213 eligible infants were enrolled and randomly allocated to group 1 (n=106; 2 + 1 schedule) or to group 2 (n=107; 1 + 1 schedule). In group 1, 91 serum samples were available for analysis 1 month after booster immunisation versus 86 in group 2. At month 13, post-booster, GMCs were equivalent between schedules for serotypes 3 (0·61 μg/mL in group 1 *vs* 0·62 μg/mL in group 2), 5 (1·74 μg/mL *vs* 2·11 μg/mL), 7F (3·98 μg/mL *vs* 3·36 μg/mL), 9V (2·34 μg/mL *vs* 2·50 μg/mL), and 19A (8·38 μg/mL *vs* 8·83 μg/mL). Infants given the 1 + 1 schedule had significantly greater immunogenicity post-booster than those given the 2 + 1 schedule for serotypes 1 (8·92 μg/mL *vs* 3·07 μg/mL), 4 (3·43 μg/mL *vs* 2·55 μg/mL), 14 (16·9 μg/mL *vs* 10·49 μg/mL), and 19F (14·76 μg/mL *vs* 11·12 μg/mL; adjusted p value range <0·001 to 0·047). The 2 + 1 schedule was superior for serotypes 6A, 6B, 18C and 23F (adjusted p value range <0·0001 to 0·017). In a predefined numerical subset of all of the infants recruited to the study (n=40 [20%]), functional serotype-specific antibody was similar between schedules. 26 serious adverse events were recorded in 21 (10%) infants across the study period; 18 (n=13) were in the 2 + 1 group and eight (n=8) in the 1 + 1 group. Only one serious adverse event, a high temperature and refusal to feed after the first vaccination visit in a child on the 2+1 schedule was considered related to vaccine.

**Interpretation:**

Our findings show that for nine of the 13 serotypes in PCV13, post-booster responses in infants primed with a single dose are equivalent or superior to those seen following the standard UK 2 + 1 schedule. Introducing a 1 + 1 schedule in countries with a mature PCV programme and established herd immunity is likely to maintain population control of vaccine-type pneumococcal disease.

**Funding:**

NIHR and the Bill & Melinda Gates Foundation.

## Introduction

A seven-valent pneumococcal conjugate vaccine (PCV7) providing protection against the most common serotypes causing pneumococcal disease in children in high-income countries was licensed in 2000. The licensing schedule consisted of three priming doses at 2, 4, and 6 months of age and a booster dose at 12 to 15 months (3 + 1 schedule). In the USA, PCV7 was introduced soon after licensure in a 3 + 1 schedule and showed both direct and indirect protection.^1^, ^2^ In the UK, PCV7 was introduced with a two-dose primary series, at 2 and 4 months of age, and a booster dose at 13 months (2 + 1 schedule). This introduction followed a pivotal immunogenicity study suggesting that 2 + 1 was likely to be effective in the UK setting.^3^ The advantage of reducing the primary schedule from three to two doses before the booster included space in the infant immunisation for additional vaccines and reduced costs. The 2 + 1 schedule proved highly effective at reducing pneumococcal disease in immunised children in the UK caused by the seven serotypes in the vaccine as well as a reduction in invasive pneumococcal disease in the unvaccinated population.^4^ A 2 + 1 PCV schedule is now used in 57 countries around the world. In 2010 the UK, together with many other countries using PCV7, switched from a seven to a 13 valent PCV (PCV13, Prevenar13; Pfizer, New York, NY, USA) with a consequent reduction in overall vaccine type invasive pneumococcal disease due to additional serotypes in PCV13 in vaccine-eligible children as well as older unvaccinated age groups.^5^

**Research in context**

**Evidence before this study**

Pneumococcal conjugate vaccine (PCV) was originally licensed as a 3 + 1 schedule and then as a 3 + 0 schedule following pivotal efficacy studies. Immunogenicity studies of a 2 + 1 schedule resulted in the use of this schedule in national immunisation programmes. Evidence subsequently accumulated of the efficacy of a 2 + 1 schedule. To assess whether fewer doses of PCV could be used without compromising efficacy we did a systematic literature review of 14 databases (Embase, PubMed, Biological Abstracts [BA], Pascal Biomed, Global Health, BioAbst/Reports, Reviews, Meetings, Cochrane Library, African Index Medicus [AIM], Western Region Index Medicus [WPRIM], Index Medicus for Eastern Med Region [MEMR], Index Medicus for South-East Asia Region [IMSEAR], Latin America and Caribbean Health Sciences Info [LILACS], Pan-American Health Organisation [PAHO], and IndiaMed [IndMed]), to identify studies that assessed alternative reduced pneumococcal vaccine schedules, published in English from Jan 1, 2010, to Dec 31, 2016, with ad-hoc additions through September, 2017. We found no other studies assessing a single infant dose of PCV followed by a booster in the second year of life.

**Added value of this study**

This immunogenicity trial, the first of its kind to study responses to a booster dose of PCV after just a single priming dose (1 + 1), has shown that responses to most serotypes in PCV13 following a booster dose at 12 months of age are similar or superior to those after a 2 + 1 schedule. This immunogenicity study lays the foundation for a move to a reduced dose 1 + 1 schedule in countries where a mature PCV programme is in place, coverage is high, and vaccine type disease is well controlled.

**Implications of all the available evidence**

Further studies of 1 + 1 schedules in low and middle income countries are currently underway. These, together with invasive pneumococcal disease surveillance and carriage studies will provide a comprehensive evidence base for future decisions about global PCV immunisation strategies. The advantages of reducing the number of doses of PCV from three to two in the infant immunisation schedule include making space in the infant programme for additional vaccines in development and potentially reduced costs. PCVs are the most expensive vaccines in infant schedules and pressure to reduce costs is found universally. Costs saved could be used to purchase other lifesaving vaccines.

With the licensure of meningococcal B vaccine (Bexsero, GSK, Rixensart, Belgium) and the decision to introduce it into the UK infant immunisation schedule, there has been a need to seek ways to reduce the number of doses, and overall cost, of vaccines delivered in infancy without compromising disease control. One option would be to reduce PCV13 to a 1 + 1 schedule, giving one priming dose at 3 months of age. Although the immunogenicity of one dose in infancy is expected to be lower than two doses, findings of a UK study with PCV9 showed higher booster responses with fewer priming doses,^3^ a finding that is well documented for meningococcal serogroup C conjugate vaccine.^6^ With the greatly reduced incidence of PCV13 disease in young children in the UK, and effective herd protection, the potentially increased risk of an invasive pneumococcal infection between 4 and 12 months of age in a child who has only received one dose of PCV13 instead of two will be very low. For example, the incidence of invasive pneumococcal disease in children younger than 2 years due to one of the seven serotypes covered by PCV7 was only 0·38 per 100 000 in 2013/14 epidemiological year^5^ compared with a pre-PCV7 incidence of 39·05 per 100 000, a 99% reduction. Invasive pneumococcal disease due to the additional six serotypes covered by PCV13 achieved an 89% reduction in incidence in children younger than 2 years within 4 years of PCV introduction, from 12·67 in 2008/10 to 1·43 in 2013/14.^5^ Mathematical models have predicted the near elimination of disease due to vaccine serotypes within the next few years^7^ although the experience with PCV13 suggests that some serotypes might be harder to eliminate.

Recent evidence from Australia suggests that in high-income settings, the sustained effect of PCVs is driven mostly by the booster dose of PCV provided in the second year of life.^8^ The booster dose contributes to the prevention of acquisition of pneumococci in the nasopharynx and hence interrupts transmission which impacts on all susceptible individuals including those in the first year of life. If one dose of PCV in the first year of life adequately primed for a booster response, it might be possible to reduce the PCV priming schedule without compromising the herd protection afforded by PCV to the unvaccinated population.

We designed a trial to compare the response to a booster dose of PCV13 when primed with a single PCV13 dose at 3 months of age compared with the standard UK immunisation schedule, where PCV is administered at 2 and 4 months of age with a booster at 12 months.

## Methods

### Study design and participants

In this multicentre, parallel group randomised controlled clinical trial, we recruited infants aged between 7 and 12 weeks with no contraindications for vaccination, as defined by Department of Health guidelines. Recruitment was by information booklets mailed out via the NHS Child Health Information Service and the UK National Health Application and Infrastructure Services, which is the agency responsible for the central NHS patient database, and via general practices in Gloucestershire and Hertfordshire that are part of the National Institute for Health Research Network.

Eligible infants had not received any other vaccinations (with the exceptions of BCG and hepatitis B). Information about the participants' past medical, medication, and vaccination history was collected either by a study paediatrician or from the medical records using a standardised case report form. Information about maternal vaccination during pregnancy with a combined tetanus, diphtheria, acellular pertussis, inactivated polio vaccine (TdaP/IPV) was based on mothers' recall. Written informed consent was obtained from parents before enrolment. Ethical approval was obtained from the Oxfordshire Research Ethics Committee (reference number 15/SC/0387).

### Randomisation and masking

Infants were randomly assigned (1:1) to receive PCV13 at 2, 4, and 12 months (2 + 1 schedule) or at 3 and 12 months of age (1 + 1 schedule) with block randomisation (block size six). Masking was not done for participants and clinical trial staff but individuals were allocated to a study number with its allocated group sequentially. Samples were received in the laboratory coded with no access to individual sample identities.

### Procedures

The two trial groups differed according to pneumococcal conjugate vaccine given. Additionally, all participants received routine vaccinations according to the UK schedule: combined diphtheria, tetanus, acellular pertussis, *Haemophilus influenzae* type b, and inactivated polio vaccine (Infanrix-IPV-Hib, GlaxoSmithKline, Rixensart, Belgium) at 2, 3 and 4 months, a meningococcal B vaccine (Bexsero, GSK, Rixensart, Belgium) at 2, 4 and 12 months of age, oral rotavirus vaccine (Rotarix, GSK, Rixensart, Belgium) at 2 and 3 months of age, Men C/Hib (Menitorix, GSK, Rixensart, Belgium) at 12 months of age, and mumps, measles and rubella vaccine at 13 months of age. Participants received concomitant paracetamol at 2 and 4 months of age.

Up to 5 mL of whole blood were taken at 5 months of age (4 weeks after the second PCV13 dose for one group and 8 weeks after the single priming dose for the other) and 4 weeks after the 12 month visit. Serological analysis was done at the WHO reference laboratory for pneumococcal serology, Great Ormond Street Institute of Child Health, University College London, London, UK. Following extraction from whole blood, sera were stored at −70°C before assay for serotype-specific immunoglobulin G (IgG) to 13 vaccine-type capsular polysaccharides (1, 3, 4, 5, 6A, 6B, 7F, 9V, 14, 18C, 19A, 19F, and 23F). Sera were assayed using the WHO reference ELISA after adsorption with cell wall polysaccharide and 22F polysaccharide at a concentration of 10 μg/mL as previously described in detail.^9^ This assay is based on the original Wyeth assay used to generate the correlate of protection of 0·35 μg/mL. The lower limit of assay quantification was 0·15 μg/mL and IgG concentrations of 0·35 μg/mL or higher were considered protective.^10^ In a subset of 40 sera functional antibodies to 13 serotypes were measured in a multiplexed opsonophagocytic assay as previously described.^11^ Values are expressed as an opsonophagocytic assay titre, equivalent to the reciprocal of the serum dilution required to produce 50% killing of the relevant serotype. Nasal swabs were taken at 12 and 18 months of age to assess pneumococcal carriage but are not reported here.

### Outcomes

The primary endpoint was serotype-specific IgG concentrations measured in blood samples collected at 13 months of age. Secondary outcomes were serotype-specific IgG concentrations measured in blood samples taken at 5 months of age, proportions 0·35 μg/mL or higher of serotype-specific IgG measured in the blood samples taken at 5 and 13 months of age, serotype-specific functional antibody measured in a subset (20%) of blood samples taken at 13 months of age, and the prevalence of carriage of vaccine and non-vaccine pneumococcal serotypes at the time of booster and 6 months later (data not available yet).

Participants were observed for 15 min after each vaccination by a study doctor or nurse, for any immediate reactions. Parents completed a diary card for 5 days from the day of vaccination and recorded all local and systemic adverse events. Unsolicited adverse events and serious adverse events occurring between the day of vaccination and the subsequent visits were recorded with details verified from GP notes as required.

### Statistical analysis

The sample size target was 110 per group with an anticipated 10% loss to achieve 100 evaluable participants per group post booster. Assuming the standard deviation of responses post booster was 0·41 this was sufficient to estimate the ratio of the geometric means between the groups with a precision of 1·3 times for the 95% confidence interval. It was also sufficient to detect 1·5 times differences with more than 80% power at a 5% significance level. For concentrations and titres, results were log transformed and groups 1 and 2 compared by the Kruskal-Wallis test for post-primary responses and by normal errors regression adjusting for sex and interval to blood collection for post-booster responses. The effect of maternal immunisation within each group on concentrations was also assessed by normal errors regression in a post-hoc analysis. Geometric means for each group were calculated with 95% confidence intervals. Proportions above concentration thresholds were compared using Fisher's exact test and proportions calculated within groups with 95% exact confidence intervals. Analysis was by modified intention to treat with all individuals included by randomised group if they had a laboratory result.

This study is registered on the EudraCT clinical trial database (2015-000817-32) and ClinicalTrials.gov, number NCT02482636.

### Role of the funding source

The funders had no role in study design or the collection, analysis, interpretation, write up of the data or decision to submit the data for publication. The corresponding author had full access to all the data in the study and final responsibility to submit the data.

## Results

Between September, 2015, and June, 2016, 376 infants were assessed for eligibility. 81 infants were excluded for not meeting the inclusion criteria (n=50) or for other reasons (n=31). 213 eligible infants were enrolled and 106 were randomly allocated to group 1 (2 + 1 schedule) and 107 to group 2 (1 + 1 schedule). In group 1, 91 serum samples were available for analysis following the visit 1 month after booster immunisation versus 86 in group 2 (table 1, figure). Median age at recruitment was 60 and 59 days, respectively for the two groups and the median age at the time of the booster was 371 days for both groups. The groups were evenly matched in terms of age at booster, although the number of infants with a long interval to the post-booster blood sample was higher for group 1 (seven *vs* two >42 days). There was also a chance imbalance in sex with 60 boys in group 1 (57%) and 49 boys in group 2 (46%), with serotype-specific responses between 10 and 26% lower in boys post booster. For these reasons post booster p values comparing groups are presented from the planned analysis in which there was adjustment for sex and interval to blood sample.

**Figure:**
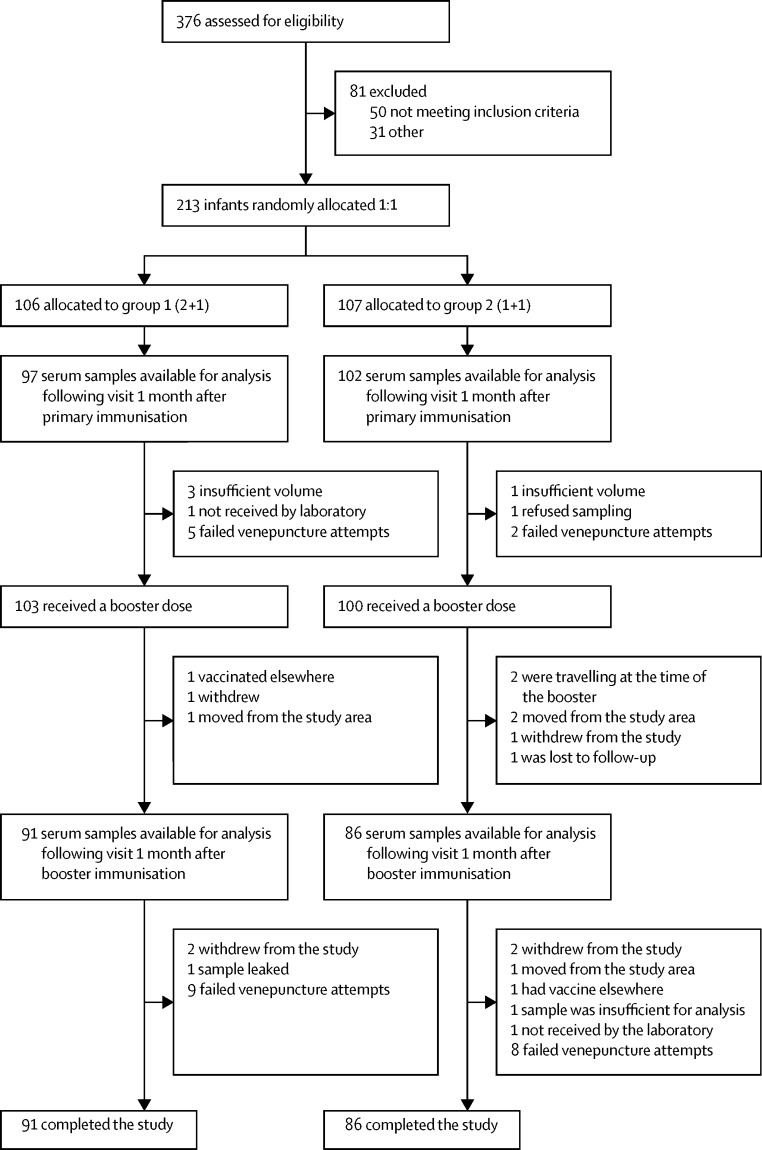
Consort details

**Table 1:** Baseline characteristics of study participants

		**Group 1 (2 + 1 schedule; N=106)**	**Group 2 (1 + 1 schedule; N=107)**
Sex			
	Male	60	49
	Female	46	58
Maternal vaccination			
	Yes	76	86
	No	23	18
	Not known	7	3
Age at first PCV dose (days)		60 (55–88)	91 (84–114)
Age at second PCV dose (group 1; days)		124 (112–154)	..
Age at booster (days)		371 (359–406)	371 (366–416)
Interval from last primary PCV dose to blood sample (days)		30 (21–57)	62 (50–92)
Interval from booster dose to blood sample (days)		30 (28–82)	20 (28–64)

Data are n or median (range). PCV=pneumococcal conjugate vaccine.

Table 2 shows the serotype-specific pneumococcal IgG values (geometric mean concentrations [GMC] in μg/mL) at 5 months of age (1 month post the primary series for group 1 and 2 months post the single priming dose for group 2), and at 13 months of age, 1 month after the booster for both groups. After the booster dose, we reported significantly higher GMCs for serotypes 1, 4, 14, and 19F in infants who received a single priming dose, while for those who received two priming doses booster responses to 6A, 6B, 18C and 23F were significantly higher. A per-protocol analysis with 24 individuals dropped because of the timing of blood sampling or vaccination being outside the recommended ranges gave similar results. When considered as proportions 0·35 μg/mL or higher, the accepted correlate of protection for PCV responses, we noted no differences post-booster between the groups since almost all vaccinees achieved this threshold (table 3). When analysing the post-booster data using serotype specific correlates previously defined by our group,^12^ comparison between the groups were similar to those with 0·35 μg/mL or higher as a cut off. Maternal immunisation status had no effect on the post booster GMCs in either group. Functional responses as measured by opsonophagocytic assay were assessed in a subset of 40 vaccinees (19 infants from group 1 and 21 from group 2) following the booster only. Geometric mean titres (GMTs) between the two groups were similar (table 4), although the study was not powered on functional responses. With the exception of serotype 4, for serotypes where IgG was significantly higher in group 1 (6A, 6B, 18C, 23F) or group 2 (1, 14, 19F), the opsonophagocytic assay GMTs showed the same trend.

**Table 2:** Post-primary and post-booster serotype specific immunoglobulin G geometric mean concentrations in μg/mL

	Post-primary group 1 (2 m, 4 m; N_max_=97)*	Post-primary group 2 (3 m; N_max_=102)*	**p value**†	Post-boost group 1 (2 m, 4 m, 12 m; N_max_=91)*	Post-boost group 2 (3 m, 12 m; N_max_=86)*	**Group 2 to group 1 ratio**‡	**Adjusted**‡**p value**
1	1·25 (1·07–1·45)	0·57 (0·47–0·69)	<0·0001	3·07 (2·58–3·64)	8·92 (7·42–10·73)	2·73 (2·13–3·51)	<0·0001
3	0·28 (0·23–0·33)	0·27 (0·21–0·34)	0·66	0·61 (0·51–0·74)	0·62 (0·52–0·74)	0·93 (0·72–1·19)	0·57
4	1·08 (0·93–1·26)	0·43 (0·36–0·51)	<0·0001	2·55 (2·15–3·04)	3·43 (2·86–4·12)	1·29 (1·01–1·64)	0·047
5	0·90 (0·77–1·07)	0·29 (0·24–0·35)	<0·0001	1·74 (1·49–2·03)	2·11 (1·81–2·45)	1·15 (0·93–1·42)	0·20
6A	1·25 (1·00–1·56)	0·13 (0·11–0·15)	<0·0001	8·62 (7·29–10·21)	6·36 (5·34–7·58)	0·69 (0·54–0·87)	0·002
6B	0·26 (0·20–0·33)	0·09 (0·08–0·09)	<0·0001	6·19 (5·10–7·50)	2·39 (1·94–2·94)	0·36 (0·27–0·47)	<0·0001
7F	2·46 (2·11–2·88)	0·81 (0·69–0·95)	<0·0001	3·98 (3·42–4·62)	3·36 (2·93–3·86)	0·82 (0·67–1·01)	0·059
9V	0·73 (0·60–0·89)	0·18 (0·16–0·21)	<0·0001	2·34 (2·00–2·73)	2·50 (2·16–2·88)	1·02 (0·83–1·26)	0·85
14	4·19 (3·23–5·43)	1·13 (0·90–1·40)	<0·0001	10·49 (8·84–12·44)	16·9 (13·54–21·08)	1·57 (1·19–2·08)	0·002
18C	0·90 (0·73–1·11)	0·22 (0·19–0·27)	<0·0001	1·98 (1·70–2·30)	1·63 (1·42–1·87)	0·78 (0·64–0·95)	0·017
19A	1·56 (1·25–1·96)	0·33 (0·27–0·39)	<0·0001	8·38 (7·17–9·80)	8·83 (7·4–10·52)	1·00 (0·79–1·26)	0·98
19F	4·54 (3·80–5·42)	0·64 (0·54–0·76)	<0·0001	11·12 (9·46–13·07)	14·76 (12·54–17·37)	1·28 (1·02–1·61)	0·035
23F	0·43 (0·34–0·54)	0·09 (0·08–0·10)	<0·0001	2·87 (2·38–3·46)	1·72 (1·44–2·05)	0·56 (0·44–0·73)	<0·0001

Data are geometric mean (95% CI).

* Table 3 shows actual numbers.

† Kruskal-Wallis test.

‡ Adjusted in regression for sex and interval to blood.

**Table 3:** Post-primary and post-booster serotype specific immunoglobulin G concentrations of 0·35 μg/mL or higher

	**Post-primary group 1 (2 m, 4 m; N_max_=97)**	**Post-primary group 2 (3 m; N_max_=102)**	p value*	**Post-boost group 1 (2 m, 4 m, 12 m; N_max_=91)**	**Post-boost group 2 (3 m, 12 m; N_max_=86)**	p value*
1	95·9% (89·8–98·9); (93/97)	74·0% (64·3–82·3); (74/100)	<0·0001	100%; (96·0–100); (91/91)	100% (95·8–100); (86/86)	n/a
3	34·5% (24·5–45·7); (29/84)	39·5% (29·2–50·7); (34/86)	0·53	75·9%; (65·5–84·4); (66/87)	78·8% (68·6–86·9); (67/85)	0·72
4	92·8% (85·7–97); (90/97)	64·4% (54·2–73·6); (65/101)	<0·0001	98·9%; (94·0–100); (90/91)	100% (95·8–100); (86/86)	1·00
5	89·6% (81·7–94·9); (86/96)	39·2% (29·7–49·4); (40/102)	<0·0001	98·9%; (94·0–100); (90/91)	100% (95·8–100); (86/86)	1·00
6A	84·4% (75·5–91); (81/96)	12·9% (7–21);(13/101)	<0·0001	100%; (96·0–100); (90/90)	100% (95·8–100); (86/86)	n/a
6B	34·0% (24·7–44·3); (33/97)	1·0% (0–5·3);(1/102)	<0·0001	100%; (96·0–100); (90/90)	97·7% (91·9–99·7); (84/86)	0·24
7F	97·9% (92·7–99·7); (95/97)	86·1%; (77·8–92·2); (87/101)	0·003	100%; (96·0–100); (91/91)	100% (95·8–100); (86/86)	n/a
9V	79·4% (70–86·9); (77/97)	16·8%; (10·1–25·6); (17/101)	<0·0001	100%; (96·0–100); (91/91)	100% (95·8–100); (86/86)	n/a
14	94·8% (88·4–98·3); (92/97)	86·3% (78–92·3); (88/102)	0·053	100%; (96·0–100); (91/91)	100% (95·8–100); (86/86)	n/a
18C	81·4% (72·3–88·6); (79/97)	33·7% (24·6–43·8); (34/101)	<0·0001	100%; (96·0–100); (91/91)	100% (95·8–100); (86/86)	n/a
19A	91·8% (84·4–96·4); (89/97)	44·1% (34·3–54·3); (45/102)	<0·0001	100%; (96·0–100); (91/91)	100% (95·8–100); (86/86)	n/a
19F	100% (96·3–100); (97/97)	79·2% (70–86·6); (80/101)	<0·0001	100%; (96·0–100); (91/91)	100% (95·8–100); (86/86)	n/a
23F	57·7% (47·3–67·7); (56/97)	5·9% (2·2–12·4); (6/102)	<0·0001	100%; (96·0–100); (91/91)	95·3% (88·5–98·7); (82/86)	0·054

Data are % (95% CI) [n/N] unless otherwise states. m=month.

* Fisher's exact test.

**Table 4:** Post-booster serotype-specific opsonphagocytic activitygeometric mean titres in a subset of infants

	**Group 1 (2 + 1 schedule; N=19)**	**Group 2 (1 + 1 schedule; N=21)**
1	132 (51–338)*	543 (267–1105)
3	108 (65–180)	144 (106–194)
4	2785 (1439–5392)	2321 (1484–3631)
5	530 (328–857)	667 (393–1130)
6A	6396 (3401–12027)	5294 (3879–7225)
6B	2088 (1224–3560)	1397 (1003–1945)
7F	3344 (2222–5034)	2953 (2353–3705)
9V	2391 (1603–3567)	1842 (1243–2731)*
14	2660 (1685–4201)	3987 (2876–5528)
18C	2783 (1710–4528)	1436 (1044–1975)
19A	3677 (1980–6829)	2915 (1982–4287)
19F	2274 (1425–3630)	2428 (1726–3415)
23F	4053 (2628–6252)	2588 (1789–3744)*

Data are geometric mean (95% CI).

* No result for one sample.

GMCs 2 months after a single dose of PCV13 at 3 months of age were significantly lower than those recorded 1 month after two doses for all serotypes except 3 (table 2). When considered as proportions 0·35 μg/mL or higher a significantly greater proportion of children receiving a single priming dose were below the correlate of protection post-primary immunisation (table 3). When analysing the data using serotype specific correlates previously defined by our group,^12^ comparison between the groups were similar to those with 0·35 μg/mL or higher as a cut off. Maternal immunisation status had no effect on the post primary GMCs in the 2 + 1 group, but, in the 1 + 1 group, post-primary GMCs were lower in infants of vaccinated mothers (range 6% lower for 6B to 62% lower for 14 and significant (5% level) for 1, 3, 4, 5, 14, 18C, and 19F.

The safety profile for the vaccine was good. 26 serious adverse events were recorded in 21 (10%) individuals across the study period: 18 (n=13) were in the 2  + 1 group and eight (n=8) in the 1 + 1 group. Only one serious adverse event, a high temperature and refusal to feed after the first vaccination visit in a child on the 2 + 1 schedule was considered related to vaccine.

## Discussion

This is the first study to formally compare booster responses to PCV after one or two priming doses of PCV provided to infants in the first year of life. Booster responses to five of the 13 serotypes were similar between the groups whereas responses to four serotypes were better in the single priming dose group compared with those receiving PCV according the UK national immunisation schedule. Responses to the remaining four serotypes, 6A, 6B, 18C and 23F, were lower in the 1 + 1 group but 1·8 (18C) to 9·2 (6B) times higher than the equivalent serotypes specific GMCs measured at 5 months of age after two priming doses. While a correlate of protection after boosting is not defined, a correlate of protection for PCV is accepted based on IgG concentrations after primary immunisation.^10^ Analysing the proportions higher than this correlate (0·35 μg/mL) in both groups after boosting showed that both achieved similar proportions higher than 0·35 μg/mL and were close to 100% for most serotypes. Enhanced boosting after fewer primary doses has been described before for meningococcal C conjugate vaccines^6^ and was shown again for some serotypes in this study. The underlying mechanism and the reason why this phenomenon only affects certain serotypes remains unclear.

As expected, the immunogenicity of a single dose at 3 months of age was significantly inferior to two doses administered at 2 and 4 months of age although it is possible that responses would have been slightly higher had they been measured at 4 rather than 8 weeks post vaccination. Despite low responses to a single dose of PCV in infancy, a single dose is associated with significant direct protection, though less than with a two or three dose priming schedule. In the USA findings of a case-control study^2^ of PCV7 showed efficacy for one dose given to infants younger than 7 months of 73% (95% CI 43–87) whereas in the UK, one dose of PCV13 given to infants younger than 12 months for the serotypes in PCV13 but not PCV7 had an efficacy of 60% (95% CI 12–82).^12^ The effect of maternal immunisation with a diphtheria toxoid-containing vaccine significantly reduced the post-primary responses to some serotypes in the 1 + 1 group in our study so the level of protection after a single dose might be less than reported previously in the absence of maternal immunisation. There was no effect of maternal immunisation on post-booster responses in either the 1 + 1 or 2 + 1 groups.

Establishing an evidence base for reducing PCV doses given during primary immunisation from two or three to a single dose followed by a booster towards the end of the first or in the second year of life, could help countries with a mature PCV programme and little vaccine type invasive pneumococcal disease to move to a reduced dose schedule. A reduction from a total of three to two PCV doses would have potentially important consequences for both specific countries making the change and the global community, by reducing the number of vaccines administered at immunisation visits and reducing the overall cost of preventing pneumococcal disease. Extended PCV vaccines have been licensed on immunogenicity alone and thus there is an established precedent for using immunogenicity studies to inform vaccine policy. An example of this was the evaluation of a 2 + 1 PCV schedule in the UK infant immunisation programme at a time when 3 + 1 was the licensed schedule.^3^ Demonstrating likely efficacy of a 2 + 1 schedule followed by implementation and evaluation of the impact of such a schedule in the UK has provided additional confidence that immunogenicity studies can adequately predict performance of PCV vaccines and has led to many countries either switching to, or introducing PCV in a 2 + 1 schedule.

While it is recognised that a single dose provides less protection against disease and carriage than a two-dose or three-dose primary series, in high-income countries with mature vaccine programmes individual protection is rarely required because the probability of exposure to vaccine-type infection is low. In England and Wales where the incidence of PCV7-type invasive pneumococcal disease in children younger than 2 years reduced by 99% within 8 years of PCV introduction,^5^ there was near elimination of PCV7 serotype carriage both in children and in older age groups.^13^ The resulting herd immunity was reflected in an overall reduction (all ages) of 86% in invasive pneumococcal disease covered by PCV7 by 2013/14.^5^ For the extra six serotypes in PCV13 (ie, those unique to PCV13 and not found in PCV7, known as PCV13-7) carriage prevalence in individuals younger than 60 years in England and Wales fell from 18·9% in 2008/09 to 0·6% by 2012/13, with an associated overall reduction in invasive pneumococcal disease covered by the PCV13-7 serotypes of 69%.^13^ Serotypes 19A and 3 were the two that accounted for the low level of persisting PCV13-7 carriage. In the USA, 7–9 years after the introduction of PCV7, there was a 97% reduction in the incidence of vaccine-type invasive pneumococcal disease in children younger than 5 years compared with the pre-PCV7 baseline^14^ while there was near elimination of nasopharyngeal carriage of PCV7 serotypes amongst healthy children in Massachusetts.^15^ Following PCV13 introduction in the USA, PCV13-7 invasive pneumococcal disease reduced by 93%^16^ and carriage of the relevant serotypes has reduced amongst children in Massachusets.^17^

Therefore, the success of a 1 + 1 PCV schedule relies on the timely provision of a booster dose and the schedule's ability to sustain indirect protection in a mature PCV programme. Toddlers and older children between the age of 12 and 36 months of age^18^ rather than infants^19^, are thought to be the major spreaders of pneumococci to their siblings and parents. Vaccinating this age group is therefore thought to be critical in establishing and sustaining indirect protection, hence reassurance is required that the booster response following a single priming dose would sustain vaccine efficacy against acquisition of carriage.^20^ As we have shown, for nine of the 13 serotypes in PCV, booster responses following a single priming dose are equivalent or superior to those after two priming doses so no reduction in the prevention of vaccine type nasopharyngeal acquisition would be anticipated. For the four serotypes where the 1 + 1 schedule led to inferior IgG responses, the lowest serotype specific correlates of protection (range 0·14–0·20 μg/mL)^12^ have been noted. Whereas at a population level less IgG is required to provide protection against infection with these serotypes, the association between circulating IgG and the prevention of carriage acquisition is unclear. The true mechanisms for preventing nasopharyngeal acquisition are not known and may be related to the local production of IgG by memory B cells resident in the nasopharynx rather than circulating IgG.^21^, ^22^

It is possible that reduced primary responses to a single dose allows continued vaccine type carriage in the first year of life before boosting. This might be especially relevant for those vaccine serotypes that persist, albeit at a low level in the community and continue to cause disease. A relevant example in the UK setting would be serotypes 3 and 19A, which continue to cause cases of invasive pneumococcal disease in all age groups. For serotype 3, no significant protection has been demonstrated in England and Wales with the 2 + 1 schedule, and invasive pneumococcal disease due to this serotype has been increasing since 2013/14.^23^ For 19A, for which there has been a major population impact, direct protection following a single dose administered below the age of 12 months was relatively low (38%, 95% CI −218 to 89).^24^ Reduced primary immunity might lead to an increase in cases in the first year of life although the extent of that increase and whether the number of expected additional cases would offset the cost savings realised by reducing the number of PCV doses administered is not clear.

While we have emphasised the importance of the booster dose in sustaining the indirect effect, in most low and middle income countries, PCV is administered according to the WHO Expanded Programme of Immunisation schedule where primary vaccination is scheduled to occur at 6, 10, and 14 weeks of age with no later booster dose (3 + 0). The long term impact of 3 + 0 schedules is difficult to gauge as few low and middle-income countries have established, high quality, population based invasive pneumococcal disease surveillance in place and sustained high coverage of PCV over a prolonged period of time. In The Gambia, where PCV7 was introduced into the routine programme in 2009 and replaced in 2011 by PCV13 and where vaccine coverage is high, the carriage prevalence of PCV7 serotypes appears to be persisting at around 20%.^25^ This persistence of carriage is in contrast to the UK and the USA where PCV7 carriage has been almost eliminated. The epidemiological characteristics of pneumococcal carriage and disease in many low-income and middle-income countries differs from that in high-income countries in a number of ways including the relatively high carriage frequencies in infants younger than 1 year^26^ and adults.^27^ It is therefore unclear what the contribution of the absence of a booster dose is to the observed persistence of PCV7 vaccine type carriage in infants in The Gambia. In high income settings it would appear from the experience in Australia that a booster dose is integral to optimising the long-term impact of PCV.^8^ However, it would be simplistic to compare the impact of PCV in high income countries with that seen in low and middle income countries and to attribute the difference in vaccine type disease control and carriage reduction solely to the presence or absence of a booster dose.

The Gavi Alliance currently supplies vaccines and vaccine support for the world's poorest countries and projects a spend on pneumococcal vaccines between 2011 and 2020 of US$4·7 billion (2016 pneumococcal AMC annual report^28^). Most countries receiving Gavi Alliance support use a 3 + 0 schedule. An eventual shift to a 1 + 1 therefore has very significant financial implications with a reduction of approximately one third in vaccine costs. This cost reduction is particularly relevant for those lower middle income countries who are soon to graduate from Gavi Alliance eligibility and will thus be required to fund their own PCV programmes. By 2020, 30 of the original GAVI Alliance eligible countries will have either graduated or be graduating and a potential one third reduction in the cost of PCV for such countries could be critical for sustaining PCV programmes. Further evaluation will be required to establish the relevance of our findings to diverse epidemiological settings.

It should be noted that this was an immunogenicity study and therefore the translation of immunogenicity findings to eventual efficacy when a 1 + 1 schedule is introduced is unclear. Furthermore, the study was done in the UK and therefore its applicability to low and middle income country settings should be interpreted with caution and ideally followed up with further immunogenicity studies in relevant settings.

In conclusions, our data suggest that booster responses following a 1 + 1 schedule are unlikely to compromise long-term control of invasive pneumococcal disease when administered in a setting where PCV coverage, particularly for the booster dose, is high at local, regional and national level, PCV has been used for some years, and pneumococcal disease and carriage of vaccine serotypes has already been controlled in all age groups. These conditions are currently likely to only be met in high-income countries and thus extrapolating this study's finding to the current situation in low and middle-income countries should be made with caution. Should a 1 + 1 schedule be considered adequate, the ideal conditions and timing favouring a switch from the current schedule still need consideration. Key to the decision to move to a 1 + 1 schedule would be acceptance of the role and importance of the indirect effect seen with conjugate vaccines. The UK Government's vaccine committee took this into account when removing the second dose of the three-dose monovalent meningococcal C conjugate vaccine schedule from infancy to adolescence, when carriage prevalence is likely to be highest.^29^ Similar recognition of the role and importance of the indirect effect will be key to underpinning any potential changes to the UK PCV infant immunisation programme. A number of randomised controlled studies comparing a 1 + 1 PCV schedule with existing immunisation schedules in India, South Africa, Vietnam, and The Gambia are currently underway. These, together with invasive pneumococcal disease surveillance and carriage studies will provide a comprehensive evidence base for future decisions about global PCV immunisation strategies.

For more on **vaccine introduction around the world** see www.view-hub.org

For the **Department of Health guidelines** see https://www.gov.uk/government/publications/contraindications-and-specialconsiderations-the-green-bookchapter-6

For the **multiplexed opsonophagocytic assay** see http://www.vaccine.uab.edu/ELISA%20Protocol.pdf

For more on **GAVI graduated or graduating countries** see www.gavi.org/about/…/gavi…/21…/14--gaviengagement-with-graduating-countries/)]
